# Edad materna avanzada como factor de riesgo de resultados adversos maternos y perinatales

**DOI:** 10.31053/1853.0605.v81.n1.41447

**Published:** 2024-03-27

**Authors:** Maria Eugenia Carducci, Gustavo Izbizky

**Affiliations:** 1 Instituto Universitario del Hospital Italiano de Buenos Aires. Hospital Italiano de Buenos Aires. Servicio de Obstetricia

**Keywords:** edad materna, complicaciones del embarazo, resultado del embarazo, atención perinatal, maternal age, pregnancy complications, pregnancy outcome, perinatal care, idade materna, complicações na gravidez, resultado da gravidez, assistência perinatal

## Abstract

**Introducción:**Se observa un progresivo aumento en la edad de las mujeres al primer embarazo, situación que se ha asociado con mayor riesgo de efectos maternos y perinatales adversos.

**Objetivo:** Describir las características y los resultados maternos y perinatales de nulíparas de 40 años y mayores y compararlos con los de nulíparas menores de 40.

**Material y métodos:**Cohorte retrospectiva de embarazos que atendieron su parto en un hospital privado universitario mediante revisión de registros.

**Resultados:**Se observó asociación entre la edad materna ≥ 40 con el resultado adverso compuesto materno (OR 1.3; IC 95%: 1,1-1,6), DBT g (OR 3,6; IC 95%: 1,8-3,7), enfermedad hipertensiva/PE (OR 2,2; IC 95%: 1,6-3,1) y hemorragia postparto (OR 4,7; IC 95%: 1,2-16,3), persistiendo la edad avanzada como factor de riesgo independiente para el resultado adverso compuesto materno (OR 1,3; IC 95%: 1,1-1,6) y perinatal (OR 1,4; IC 95%: 1,2-1,7) en el análisis multivariado. Se observó mayor tasa de parto pretérmino en el grupo de nulíparas añosas (OR 1,6; IC 95%: 1,3-2,0) con mayor requerimiento de ingreso a UCIN para sus recién nacidos (OR 1,3; IC 95%: 1,0-1,8).

**Conclusiones:**Las mujeres con edad materna avanzada constituyen una población de alto riesgo, cuya atención y seguimiento requeriría un enfoque diferencial que tenga como objetivo mejorar los resultados maternos y perinatales.

## Abstract (en)

**Introduction:** A progressive increase in the age of women at first pregnancy is observed, a situation that has been associated with a greater risk of adverse maternal and perinatal effects.

**Objective:**The aim of this study was to describe the characteristics and the maternal and perinatal outcomes of nulliparas of 40 years and older and compare them with those of nulliparas under 40.

**Study design:**This was a retrospective cohort analysis of a database of pregnancy population who had attended their deliveries in a private university hospital.

**Results:** An association was observed between maternal age ≥ 40 with the maternal compound adverse outcome (OR 1,3; 95% CI: 1,1-1,6), gestational diabetes (OR 3,6; 95% CI: 1,80-3,7), hypertensive disorders/preeclampsia (OR 2,2; 95% CI: 1,6-3,1) and postpartum hemorrhage (OR 4,7; 95% CI: 1,2-16,3), with advanced maternal age persisting as an independent risk factor for the maternal compound adverse outcome (OR 1,3; 95% CI: 1,1 -1,6) and the perinatal compound adverse outcome (OR 1,4; 95% CI: 1,2-1,7) in the multivariate analysis. A higher rate of preterm birth was observed in the group of older nulliparas (OR 1,6; 95% CI: 1,3-2,0) with a higher requirement for admission to NICU for their newborns (OR 1,3; 95% CI: 1,0-1,8).

**Conclusions:** Women with advanced maternal age constitute a high-risk population, whose attention and follow-up would require a differential approach aiming to improve maternal and perinatal outcomes.

## Resumo

**Introdução:** Observa-se um aumento progressivo da idade das mulheres à primeira gravidez, situação que tem sido associada a um maior risco de efeitos adversos maternos e perinatais.

**Objetivo:** Descrever as características e desfechos maternos e perinatais de nulíparas com 40 anos ou mais e compará-los com os de nulíparas com menos de 40 anos.

**Material e métodos:** Coorte retrospectiva de gestações que deram à luz em um hospital universitário privado por meio de revisão de prontuários.

**Resultados:** Observou-se associação entre idade materna ≥ 40 anos com o desfecho adverso composto materno (OR 1,3; IC 95%: 1,1-1,6), DBT g (OR 3,6; IC 95%: 1,8-3,7), doença hipertensiva/EP (OR 2,2; IC 95%: 1,6-3,1) e hemorragia pós-parto (OR 4,7; IC 95%: 1,2-16,3), com idade avançada persistindo como fator de risco independente para desfecho adverso composto materno (OR 1,3; IC 95%: 1,1 -1,6) e perinatal (OR 1,4; IC 95%: 1,2-1,7) na análise multivariada. Maior taxa de parto prematuro foi observada no grupo de idosas nulíparas (OR 1,6; IC 95%: 1,3-2,0) com maior necessidade de internação em UTIN para seus recém-nascidos (OR 1,3; IC 95%: 1,0-1,8).

**Conclusões:** As mulheres com idade materna avançada constituem uma população de alto risco, cujo cuidado e acompanhamento exigiriam uma abordagem diferenciada que vise melhorar os resultados maternos e perinatais.

**CONCEPTOS CLAVE**

**Qué se sabe sobre el tema.**

Durante las últimas décadas, los países desarrollados han experimentado un aumento en la edad promedio de las mujeres al momento de la concepción y el parto.

La edad materna avanzada se considera un factor de riesgo de resultados maternos y perinatales adversos. Algunas complicaciones maternas incluyen diabetes mellitus gestacional e hipertensión gestacional.

**Qué aporta este trabajo.**

El presente trabajo da muestra de las mismas tendencias y asociaciones a nivel local.

Sugerimos que el embarazo a la edad de 35 años o más se reconozca como un factor de riesgo de resultados maternos, fetales y neonatales adversos al asesorar a las pacientes y poder así determinar planes de manejo acordes.

**Divulgación**

Posponer la maternidad es un tema cada vez más importante para las mujeres y para la salud pública. Es útil en el asesoramiento de pacientes en búsqueda de embarazo, tomar conciencia sobre los riesgos especiales asociados a una edad materna avanzada. Es de suma importancia para los profesionales y para la organización del sistema de salud entender que la atención de estos embarazos requiere más apoyo social, económico y emocional que para las parejas jóvenes.

La edad materna avanzada como factor de riesgo de resultados adversos perinatales

## Introducción

Entre las tendencias sociodemográficas que se pueden verificar en salud materno infantil en las últimas décadas, se encuentra la disminución de la tasa de fertilidad (TF). Entre 1950 y 2017 disminuyó en un 49 % (de 4,7 nacidos vivos a 2,4 nacimientos vivos por cada 1000 mujeres); en tanto cuando se analiza la tasa específica de fertilidad por edad (ASFR), ésta muestra un aumento en mujeres de 30 años o más, al mismo tiempo que una disminución en mujeres jóvenes entre 20 y 24 años. Paralelamente a este fenómeno, se ha observado un incremento de la edad materna al primer embarazo, reflejándose en las tasas de primiparidad a edades más avanzadas ^1^

Si bien las tendencias no son homogéneas alrededor del mundo, existen algunos patrones como los enormes cambios en la ASFR y en la mortalidad materna, que tienen efectos sociales y económicos sustanciales, resaltando la necesidad e importancia del monitoreo y análisis de estos indicadores a nivel de la población local ^2^ .

A nivel de la región de las Américas, es en los Estados Unidos donde se verificó un aumento significativo en el número de mujeres que tienen sus bebés a partir de los 35 años; este cambio comenzó a mediados de la década de 1970 y ha continuado a lo largo de los años. Hoy en día, el 15% de las mujeres que dan a luz en ese país tienen más de 35 años, comparado con un 11% en 2002 y un 8% en 1990. De la mano de esta modificación en la edad reproductiva poblacional, vienen modificaciones en grupos específicos, como el de las mujeres que tienen su primer hijo (nulíparas). A mediados de la década de 1970 las mujeres nulíparas de 35 a 39 años representaban el 1,7 por mil embarazadas mientras que en 2012 esa cifra aumentó al 11 por 1.000, un incremento de 6 veces ^3, 4^ . A nivel mundial la prevalencia estimada de mujeres embarazadas ≥ 35 años es del 12,3% ^5^ . En nuestro país un estudio realizado por la Dirección General de Estadísticas y Censos Porteña comparó la proporción de nacimientos en diferentes grupos etarios entre 1990 y 2015, representando el 12,6% vs. el 21,7% para el grupo de 30 a 35 años y el 2,9% vs. 6,6% del total de nacimientos para mujeres de 40 años o más, respectivamente ^6^ . A nivel país las cifras reportadas por el Ministerio de Salud de la Nación indican para el año 2017 que el 17% de los nacidos vivos provienen de mujeres de 35 o más años ^7^ .

En 1958 el Consejo de la Federación Internacional de Obstetras y Ginecólogos (FIGO) ^8^ , acuñó el término "primigesta añosa" para resaltar que la edad materna (>35 años) es un factor de riesgo obstétrico y perinatal; sin embargo esta definición clásica ha quedado estrecha, debido a la aparición de nuevas categorías como "extremo de la edad reproductiva" para mujeres de 40 añoso más ^9^ y "edad materna muy avanzada" ^10-12^ para mujeres de 45 años o más, cuyos riesgos específicos recién comienzan a estudiarse.

La mayor parte de la evidencia publicada, reporta los resultados en mujeres de 35 años o más incluyendo un número pequeño de pacientes con edades más avanzadas, siendo estas últimas el grupo de mujeres en el que los resultados adversos probablemente sean más frecuentes ^13^ . El foco del presente estudio está puesto en el grupo comprendido por mujeres nulíparas de 40 años o más.

Entre los problemas reproductivos, obstétricos y perinatales descriptos en un gradiente creciente a medida que aumenta la edad materna ^14^ se encuentran: la reducción de la fertilidad ^14^ , mayor frecuencia de ciertas anomalías cromosómicas ^15^ ; diabetes gestacional (DBT g) ^9^ , trastornos hipertensivos y preeclampsia (PE) ^8, 10^ , aborto espontáneo, anomalías del crecimiento fetal, hemorragia postparto, desprendimiento de placenta normoinserta (DPNI) ^8^ , parto pretérmino y mayor tasa de finalización por cesárea ^8,11, 12^ ; embarazo múltiple ^16^ ; mayor tasa de admisión de recién nacidos a cuidados intensivos neonatales (UCIN) ^17^ .

Dado que las mujeres embarazadas con edad materna avanzada constituyen una parte creciente de la población gestante y dado que presentan desafíos particulares en cuanto al cuidado médico, creemos que es relevante conocer en profundidad las características de este grupo poblacional y contar con información a nivel local para poder así optimizar las estrategias de cuidado y mejorar los resultados gestacionales. Por ello, el objetivo de este estudio fue caracterizar el riesgo materno y neonatal asociado con la nuliparidad en el extremo superior de la edad reproductiva (40 años o más).

## Materiales y Métodos

Se realizó un estudio de cohorte retrospectiva de nacimientos ocurridos en el Hospital Italiano de Buenos Aires en el período comprendido entre enero 2014 a agosto de 2017. Se incluyeron todos los nacimientos consecutivos de madres nulíparas, con embarazos mayores a 24 semanas, con feto vivo o muerto, que tuvieran seguimiento del embarazo y parto en la Institución. Se excluyeron embarazos con malformaciones congénitas mayores.

La cohorte se estratificó en dos grupos de edad como factor de exposición: nulíparas < 40 años y nulíparas ≥ 40 años y se compararon las características maternas y los resultados obstétricos y perinatales. A los efectos de este estudio, se definió como resultado primario compuesto materno a la presencia de cualquiera de los siguientes problemas o más de uno: enfermedad hipertensiva, preeclampsia, restricción de crecimiento intrauterino (RCIU), DBT g, DPNI, hemorragia postparto y evento trombótico.

A los efectos de este estudio, se definió como resultado primario compuesto feto-neonatal a la presencia de cualquiera de los siguientes problemas o más de uno: muerte fetal, admisión a la Unidad de Cuidados Intensivo Neonatales (UCIN), depresión neonatal, parto pretérmino y bajo peso al nacer.

Se estimó en al menos 610 mujeres el tamaño muestral necesario, considerando una frecuencia de un resultado adverso compuesto (enfermedad hipertensiva, PE, RCIU y DBT g) de alrededor del 15% en el grupo de menores de 40 años para un riesgo relativo de 1,5 con un poder del 80% y un nivel de significación de 0,05.

Las variables categóricas se analizaron por el método de Chi cuadrado con corrección de Fisher cuando fue necesario. Las variables interválicas se describieron con media o mediana según su distribución y sus respectivas medidas de dispersión (desvío estándar - IIC). Para la comparación de variables continuas se utilizó el test t de Student o el Wilcoxon rank sum test, de acuerdo a la distribución expresada. La asociación entre variables fue descripta mediante razones de probabilidad (Odds Ratios), con sus respectivos intervalos de confianza del 95%.

En el análisis multivariado se utilizó el modelo de regresión logística. Se incluyeron aquellas variables independientes con significación estadística (p < 0,05) en el análisis univariado.

El presente estudio fue aprobado por el Comité de Ética de Protocolos de Investigación del Hospital Italiano de Buenos Aires (Protocolo Nº 3117, fecha de aprobación 23/2/2017).

## Resultados

Durante el período de estudio se atendieron en la Institución un total de 4095 mujeres nulíparas que cumplían todos los criterios de inclusión y no presentaban criterios de exclusión (Figura 1). La prevalencia de nulíparas añosas fue de 15,4% (n=634) con una edad media de 42 ± 2,37 años. De estas mujeres, el 16% (n=101) correspondió a un subgrupo de embarazadas ≥ 45 años. En el grupo de las mujeres < 40 años la media de edad fue de 31 ± 5 años. La distribución por grupo etario durante los años que comprendió el estudio se observan en la Figura 2.

**Figura Nº 1:**
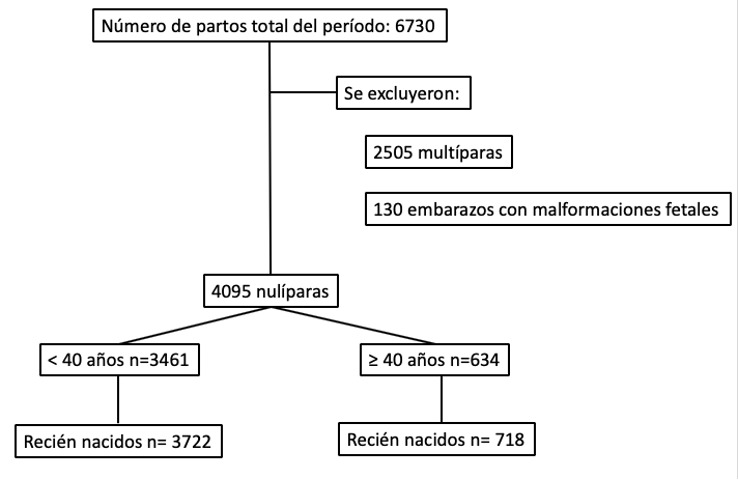
Diagrama de flujo de pacientes incluidas en el estudio

**Figura Nº 2.**
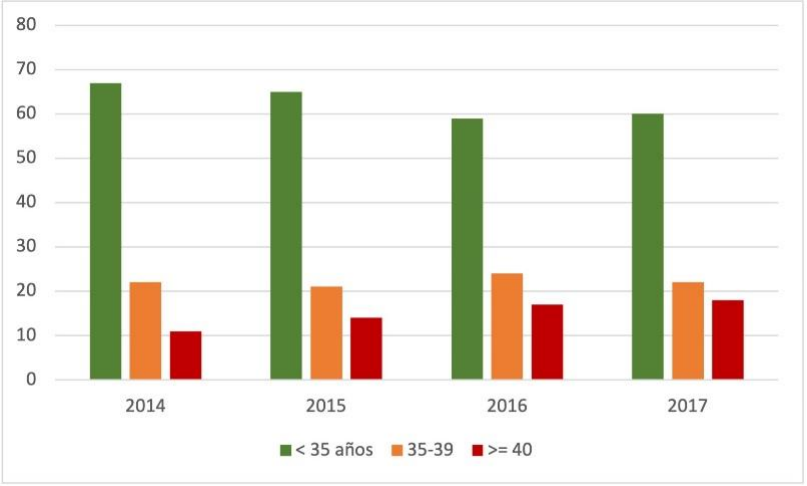
Distribución de embarazadas por grupo etario durante los años que comprendió el estudio

En la Tabla 1 se describen las características demográficas de la población de estudio. El promedio de peso corporal fue de 64 ± 12 kg. para las <40 años y 66 ± 13 Kg (p= 0.0032) para las mayores. En la tabla se puede observar la distribución de acuerdo al índice de masa corporal (IMC) con diferencia significativa para sobrepeso y obesidad en el grupo de mujeres añosas (P =0,05). La tasa de enfermedad materna de base (crónica) fue mayor en las mujeres ≥ 40 años (OR 1,50; IC 95%: 1,0-2,0). Con respecto a la forma de concepción, la tasa de embarazos logrados por técnicas de reproducción asistida mostró un OR 6,7 (IC 95%: 5,6-8,1) para las añosas mientras que la frecuencia de embarazos múltiples fue casi el doble para este grupo (OR 1,9; IC 95%: 1,4-2,5).

**Tabla Nº 1:** Características demográficas de la población.

**Características Sociodemográficas**	**Mujeres < 40 años**		**Mujeres > 40 años**		**p/OR (IC 95%)**
	**(n=3641)**		**(n=634)**		
	**n**	**%**	**n**	**%**	
**Índice de masa corporal**					
< 25	2198	63,4	370	58,6	
25-29.9	850	24,6	172	27,2	
≥ 30	413	12	90	14,2	0,05
**Tabaco**					
No fumadora	3292	95,1	606	95,9	
Abandonó	137	4	16	2,5	
Fumó durante embarazo	32	0,9	10	1,6	0,07
**Enfermedad Materna**					
No	3247	94	576	91,1	
Si	209	6	56	8,9	6,7 (5,6-8,0)
**Forma de concepción**					
Concepción espontánea	2738	79,9	228	36,1	
Tratamiento de fertilidad	723	20,1	404	63,9	
Baja complejidad	101	14	0	0	
Alta complejidad propios	556	76	234	58	
Ovodonación	66	10	170	42	6,1 (4,3-8,5)
**Embarazo múltiple**					
Si	261	727	84	1329	1,9 (1,4-2,5)

Se constató que en el grupo de mujeres ≥ 40 existe una fuerte tendencia a la terminación electiva de la gestación (inicio espontáneo del trabajo de parto 28% versus 56% para las jóvenes, p<0,001), mientras que el inicio inducido fue similar para ambos grupos (19% versus 14%). Las mujeres ≥ de 40 años mostraron una proporción mayor de intervención al momento del parto (73,9% vs 46,3%, OR 3,2; IC 95%: 2,7-3,9). La proporción de cesáreas (Tabla 2) fue significativamente mayor en el grupo de ≥ 40 años (OR 3,7; IC 95%: 2,9-4,5). En la Tabla 2 se observa que la edad materna avanzada en el análisis univariado mostró asociación con el resultado adverso compuesto materno (OR 1,3; IC 95%: 1,1-1,6), DBT g (OR 3,6; IC 95%: 1,8-3,7), enfermedad hipertensiva/PE (OR 2,2; IC 95%: 1,6-3,1) y hemorragia postparto (OR 4,7; IC 95%: 4,7-16,3)

**Tabla Nº2:** Resultados maternos.

	**Mujeres < 40 años**		**Mujeres ≥ 40 años**			
**Resultados obstétricos**	**(n=3461)**		**(n=634)**			
	**n**	**%**	**n**	**%**	**OR (IC 95%)**	
Resultado primario compuesto*	**808**	**22,5**	**182**	**28,7**	**1,3 (1,1-1,6)**	
**EHE/PE**	**137**	**3,8**	**52**	**8,2**	**2,2 (1,6-3,1)**	
**DBT g**	**29**	**0,8**	**16**	**2,8**	**3,6 (1,8-3,7)**	
**RCIU**	**339**	**9,4**	**70**	**9,8**	**1,0 (0,7-1,3)**	
**Placenta previa**	**12**	**0,3**	**4**	**0,6**	**1,9 (0,4-6,2)**	
**DPNI**	**14**	**0,4**	**2**	**0,3**	**0,8 (0,08-3,5)**	
**Hemorragia postparto**	**7**	**0,2**	**6**	**0,9**	**4,7 (1,2-16,3)**	
**Evento trombótico**	**0**	**0,0**	**0**	**0,0**		
**Parto vaginal**	**1578**	**46,0**	**118**	**18,3**		
**Cesárea**	**1883**	**54,0**	**516**	**81,7**	**3,7 (2,9-4,5)**	
**Inducción al t de p/electivo**	**1530**	**44,2**	**450**	**7,8**	**3,0 (2,5-3,7)**	

*EHE: Enfermedad hipertensiva del embarazo. PE: Preeclampsia. DBT g: Diabetes gestacional. RCIU: Restricción de crecimiento intrauterino. DPNI: Desprendimiento prematuro de placenta normoinserta.

En el análisis multivariado la edad materna mayor de 40 años se asoció de manera independiente con el resultado adverso compuesto materno (OR 1,3; IC 95%: 1,1-1,6). Otras variables que persistieron como factores de riesgo independientes luego del análisis multivariado fueron el sobrepeso (OR 1,4; IC 95%: 1,1-1,7), la obesidad (OR 1,6; IC 95%: 1,2-19), el embarazo múltiple (OR 11,0; IC 95%: 8,0-15,0), y la presencia de enfermedades maternas crónicas (OR 2,3; IC 95%: 1,8-3,0). Un hallazgo inesperado fue la asociación negativa entre los procedimientos de fertilización asistida de alta complejidad y el resultado compuesto (OR 0,6; IC 95%: 0,5-0,8).

En la Tabla 3se muestran los resultados perinatales. En el análisis multivariado la edad materna mayor de 40 años se asoció de manera independiente con el resultado adverso compuesto perinatal (OR 1,4; IC 95%: 1,2-1,7). Se observó mayor tasa de parto pretérmino en el grupo de nulíparas añosas (OR 1,6; IC 95%: 1,3-2,0) con mayor requerimiento de ingreso a UCIN para sus recién nacidos (OR 1,3; IC 95%: 1,0-1,7). Por otra parte los factores que se asociaron en forma estadísticamente significativa a este resultado en forma independiente fueron el embarazo múltiple (OR 21,4; IC 95%: 15,1-30,4), la presencia de enfermedades maternas crónicas (OR 2,4; IC 95%: 1,8-3,3) y la obesidad (OR 1,2 IC 95% 1,0-1,5). Por último se encontró diferencia en el peso al nacer a favor de las más jóvenes, 3182 ± 642 gramos versus 3020 ± 679 gramos (p=0,001) al igual que mayor edad gestacional al nacimiento 38.4 ± 2.2 semanas versus 37.7 ± 2.5 semanas.

**Tabla Nº 3:** Resultados perinatales en nulíparas añosas y < 40 años.

	**Mujeres < 40 años**		**Mujeres ≥ 40 años**		**OR (IC 95%)**	
**Resultados perinatales**	**(n=3722)**		**(n=718)**			
	**n**	**%**	**n**	**%**		
Resultado perinatal compuesto*	740	19,9	190	26,6	1,40 (1,20-1,70)	
Admisión a UCIN (n - %)	338	9,1	86	12,0	1,30 (1,04-1,77)	
Depresión al min de vida**	66	1,8	10	1,4	1,10 (0,50-2,20)	
Depresión severa a los 5 min**	6	0,17	0	0,0		
Tasa de parto pretérmino	454	12,2	132	18,4	1,60 (1,31-2,03)	
% Parto pretérmino iatrogénico	306	67,6	114	86,0	1,20 (0,90-1,60)	
% Parto pretérmino espontáneo	148	32,4	18	14,0	1,30 (0,70-2,20)	
RCIU	349	9,4	70	9,8	1,0 (0,70-1,30)	
Muerte fetal	17	0,47	2	0,3	0,60 (0,06-2,40)	

*(Muerte fetal, admisión a la Unidad de Cuidados Intensivo Neonatales (UCIN), depresión neonatal, parto pretérmino y bajo peso al nacer).

**(Apgar ≤ 3)

## Discusión

El presente estudio encontró que la edad materna avanzada en nulíparas es un factor de riesgo independiente para resultados adversos maternos y perinatales.

Una de las características destacables encontradas en la población en estudio es la prevalencia elevada de nulíparas añosas del 15,4%, siendo esta cifra mayor que la reportada a nivel país, donde la tasa global de mujeres mayores de 40 años con nacidos vivos es de 3,5% ^18^ , por lo tanto la tasa de nulíparas de esta edad a nivel país debe ser mucho menor, teniendo en cuenta que es más probable que esas mujeres sean en su mayor parte multíparas. La prevalencia en nuestra muestra es alta, aún cuando se la compara con la reportada en otros países o regiones: 3,9% en California ^19^ 4,6% en Alemania ^20^ , 2% Taiwan ^21^ . A nivel global la elevada prevalencia de nulíparas añosas podría ser resultado de diferentes fenómenos como los cambios en el estilo de vida relacionados con mayores oportunidades en el campo de la educación, mayor presencia de mujeres en el campo laboral, mejoras en materia de legislación de protección de la maternidad, cambios en valores, la equidad de género y la efectividad de los métodos de control de la natalidad, el aumento en la expectativa de vida ^22, 23^ entre otros. Esto explica sobre todo la tendencia en zonas urbanas donde el acceso a la educación y a los puestos de trabajo en grandes empresas es mayor, al igual que la disponibilidad de las técnicas de reproducción asistida. Al ver lo que ocurre con la prevalencia a nivel local, podemos plantear como posibles explicaciones que el estudio se realizó en una población cerrada de nivel socioeconómico medio donde la mayoría de las mujeres alcanza un nivel de educación superior con mayor acceso a la anticoncepción y puestos laborales con pobre flexibilidad para las mujeres que además son madres. La población asistida en el Centro donde se realizó el estudio se ajusta mayormente a la definición de clase media que se sitúa entre los deciles 3 y 9 en la línea de ingresos según la CEPAL ^24^ . Otro factor social que podría influir en este resultado es la mayor frecuencia de divorcios lo que plantea el reinicio de nuevas parejas y retrasa muchas veces la maternidad. Por último, los tratamientos de fertilidad que como se mencionó previamente permiten hoy embarazos en edades maternas en las que biológicamente la probabilidad de lograr un embarazo espontáneo es casi nula.

Como se hipotetizó, la tasa de enfermedad hipertensiva gestacional y PE fue mayor en el grupo de estudio. Esto podría tener relación con la edad materna por sí sola, pero también podría asociarse a la mayor prevalencia de hipertensión crónica y a la mayor tasa de ovodonación observadas en este grupo, siendo este último un factor de riesgo independiente ya descripto por diversos autores para trastornos hipertensivos ^17,25, 26^ .

La edad materna avanzada también mostró asociación significativa con la frecuencia de hemorragia postparto en coincidencia con lo reportado por otros autores. Ben-David y col. reportan una tasa de hemorragia postparto de 2,7% para el subgrupo ≥ 45 años ^10^ , mientras que Sheen y col. reportan una tasa de entre 2,5 y 3% para mujeres de 40-44 años y aún mayor (4.8%) para el grupo de 45 años y mayores ^27^ . La mayor frecuencia de este evento podría tener correlación con que las mujeres de mayor edad tienen mayor prevalencia de miomatosis uterina, mayor infiltración grasa del útero, y mayor frecuencia de adenomiosis, pudiendo representar todas estas patologías uterinas una mayor dificultad para la adecuada retracción uterina postparto.

La edad materna avanzada mostró ser un factor de riesgo independiente para el resultado adverso compuesto materno (OR 1,3; IC 95%: 1,1-1,6).

No se observaron diferencias para la tasa de RCIU, placenta previa, DPNI, muerte fetal y depresión neonatal entre ambos grupos. Por otro lado, probablemente por lo infrecuente del evento, no se observó durante el período de estudio ningún episodio de trombosis intraembarazo.

En coincidencia con estudios previamente publicados, nuestros datos muestran que la cesárea es el modo de nacer más prevalente en este grupo etario, siendo la mayoría realizadas en forma electiva. Esto puede atribuirse al deseo materno de tener un parto seguro y programado ya que la mayoría de estas pacientes no tendrán nuevos embarazos ^4, 28^ . Esta tendencia de finalización electiva, muchas veces sin justificación médica, tiene consecuencias como el aumento del número de nacimientos de "término temprano", definido como aquel nacimiento entre la semana 37 y 38,6 de gestación que se asocia a un aumento de la mortalidad neonatal, y de la morbilidad en complicaciones como síndrome de distrés respiratorio, taquipnea transitoria, sepsis e ingreso a unidad de cuidados intensivos neonatales ^29, 30^ . Nuestros datos, apoyan la existencia de este sesgo ya que las mujeres de más edad tienen una edad gestacional al nacimiento menor que las jóvenes y con mayor prevalencia de resultado compuesto adverso perinatal.

En cuanto a la tasa de parto pretérmino, además de haber sido más elevada para el grupo de nulíparas añosas, se pudo observar en esta población que se trató en su mayoría de un parto pretérmino iatrogénico (86,3%). Esto probablemente encuentre explicación por la mayor tasa de complicaciones obstétricas observadas en este grupo, información que también concuerda con la literatura internacional.

El presente estudio presenta como principal fortaleza el tamaño muestral, además de contar con la información del seguimiento durante el embarazo, postparto y los resultados neonatales de una población local.

Como principales limitaciones se trata de una población cerrada, atendida en un Hospital terciario de alta complejidad, de nivel socioeconómico medio, la mayoría con alto nivel de escolarización y un elevado cumplimiento de control prenatal, con una proporción de embarazos gemelares elevada por contar con una clínica de seguimiento de embarazo gemelar que recibe un gran número de derivaciones además de controlar la población local. Esto podría afectar la validez externa de los hallazgos, aunque son coincidentes con la literatura.

Por otra parte, el tipo de diseño hace al estudio susceptible del sesgo de información aunque consideramos que pudo haber sido minimizado por el hecho de contar con registros de historia clínica electrónica.

## Conclusiones

El presente trabajo muestra que una importante proporción de mujeres que buscan embarazo en edades avanzadas son nulíparas, y las mismas representan una población de alto riesgo que merece una visión y una estrategia de cuidado diferente.

La edad materna avanzada debería considerarse un problema de salud pública emergente, ya que las mujeres en este contexto tienen mayor morbilidad y mayor número de embarazos múltiples. Como cualquier población de alto riesgo, su atención y seguimiento representa un desafío que pareciera requerir unidades especializadas en la atención de este tipo de embarazos, con un enfoque diferencial que tenga como objetivo mejorar los resultados maternos y perinatales.
